# Chemical Constituents from the Fruits of *Forsythia suspensa* and Their Antimicrobial Activity

**DOI:** 10.1155/2014/304830

**Published:** 2014-03-12

**Authors:** Ping-Chung Kuo, Guo-Feng Chen, Mei-Lin Yang, Ya-Hua Lin, Chi-Chung Peng

**Affiliations:** ^1^Department of Biotechnology, National Formosa University, Yunlin 632, Taiwan; ^2^Department of Chemistry, National Chung-Hsing University, Taichung 402, Taiwan

## Abstract

Lignans and phenylethanoid glycosides purified from *Forsythia suspensa* were reported to display various bioactivities in the previous literature, including the antimicrobial activity. Therefore, the present research is aimed to purify and identify the chemical constituents of the methanol extracts of fruits of *F. suspensa*. The methanol extracts of fruits of *F. suspensa* were fractionated and further purified with the assistance of column chromatography to afford totally thirty-four compounds. Among these isolates, 3**β**-acetoxy-20**α**-hydroxyursan-28-oic acid (**1**) was reported from the natural sources for the first time. Some of the purified principles were subjected to the antimicrobial activity examinations against *Escherichia coli* to explore new natural lead compounds.

## 1. Introduction

Food safety is an important public health issue continuously attracting researchers from various fields. The use of biopreservatives and pathogen antagonists had been completed as a means of protecting the microbiological safety of fresh and processed products [1–4]. Lignans and phenylethanoid glycosides are widely distributed among plant bioresources and those purified from* Forsythia suspensa* have already been reported to exhibit antimicrobial bioactivities in the previous literature [5–13]. Although these natural compounds did not exhibit better inhibition of the bacterial growth, they were not very toxic while compared with the synthetic antibiotics.* F. suspensa* (Oleaceae) is an important original plant of the crude drug “rengyo” (Forsythiae Fructus) which has been used for anti-inflammatory, diuretic, drainage, and antimicrobial purposes in Oriental medicine [6, 8]. Previous phytochemical investigations of* Forsythia* genus afforded a series of steroids, triterpenoids, lignans, and phenylethanoid glycosides [5–16]. In our continuous program aimed to the bioactive principles from natural sources, the fruits of* F. suspensa* were selected as the target due to their antimicrobial potential in our preliminary bioassay (Table 1). In the present study, we wished to report the structural characterization of one new triterpene, 3*β*-acetoxy-20*α*-hydroxyursan-28-oic acid (**1**), along with thirty-three known compounds, as well as their antimicrobial effects against* E. coli*. We hoped to explore new lead compounds which could be performed for further investigation of the new antibiotic agents.

## 2. Materials and Methods

### 2.1. General Procedure

Melting point was determined by a Fisher Scientific melting point measuring apparatus without corrections. The IR spectrum was obtained, as a KBr disc, on a Bruker Tensor 27 FT-IR spectrometer. Optical rotation was measured with an Atago AP-300 automatic polarimeter. ^1^H- and ^13^C-NMR, COSY, HMQC, HMBC, and NOESY spectra were recorded on the Varian Unity 400 and Bruker AV 500 NMR spectrometers, using tetramethylsilane (TMS) as the internal standard. Standard pulse sequences and parameters were used for the NMR experiments and all chemical shifts were reported in parts per million (ppm, *δ*). The low and high-resolution FAB mass spectra were obtained on a JEOL JMS-700 spectrometer operated in the positive-ion mode. All the chemicals were purchased from Merck KGaA (Darmstadt, Germany) unless specifically indicated. Column chromatography was performed on silica gels (Kieselgel 60, 70–230 mesh, Merck KGaA). Thin layer chromatography (TLC) was conducted on precoated Kieselgel 60 F 254 plates (Merck) and the compounds were visualized by UV light or spraying with 10% (v/v) H_2_SO_4_ followed by heating at 110°C for 10 min.

### 2.2. Plant Materials

The fruits of* Forsythia suspensa* were purchased from the herbal markets in Yunlin, Taiwan, and authenticated by Dr. C. S. Kuoh (Department of Bioscience, National Cheng Kung University, Tainan, Taiwan). A voucher specimen (PCKuo_2007001) was deposited in the herbarium of the Department of Biotechnology, National Formosa University, Yunlin, Taiwan.

### 2.3. Extraction and Isolation

The fruits of* Forsythia suspensa* (6.0 Kg) were powdered and refluxed with methanol (20 L × 7), and the combined extracts were concentrated under reduced pressure to give a brown syrup (1.4 Kg). The crude extract was suspended into water and partitioned with chloroform, successively to afford chloroform (450 g) and water soluble fractions (950 g), respectively.

The chloroform soluble extracts were purified by silica gel column chromatography (SiO_2_ CC) eluted with* n*-hexane and acetone gradients (100 : 1 to 1 : 1) to afford 8 fractions as monitored by TLC. Fractions 4, 5, and 8 display significant spots and therefore were subjected to the further purification. Fraction 4 was purified by SiO_2_ CC eluted with* n*-hexane/ethyl acetate (50 : 1) to yield three subfractions (F4.1~4.3). The subfraction F4.2 displayed significant spots and was applied to SiO_2_ CC, eluted with* n*-hexane and acetone gradients (100 : 1 to 1 : 1), to afford *β*-amyrin acetate (**2**) (10 mg) and taraxasterol acetate (**3**) (6 mg). The subfraction F4.3 was purified with SiO_2_ CC eluted with* n*-hexane and acetone gradients (300 : 1 to 1 : 1) to yield three minor fractions (F4.3.1~4.3.3). The minor fraction F4.3.1 was further applied to SiO_2_ CC with benzene : ethyl acetate (50 : 1) solvent system to afford 3*β*-acetyl-20,25-epoxy-dammarane-24*α*-ol (**4**) (25 mg). F4.3.2 was repeatedly subjected to SiO_2_ CC and preparative TLC (pTLC) (eluted with benzene : acetone, 20 : 1) to yield 3*β*-acetoxy-20*α*-hydroxyursan-28-oic acid (**1**) (10 mg). F4.3.3 was recrystallized with acetone to produce acetyl oleanolic acid (**5**) (20 mg). Fraction 5 was purified by SiO_2_ CC eluted with* n*-hexane/ethyl acetate (50 : 1) to yield ten subfractions (F5.1~5.10). Subfractions F5.5, 5.6, 5.8, and 5.10 were major fractions and displayed significant spots by TLC monitoring. F5.5 was further isolated by SiO_2_ CC with a mixed eluent of benzene and acetone (200 : 1) to afford 3*β*-acetyl-20,25-epoxy-dammarane-24*α*-ol (**4**) (20 mg). F5.6 was also subjected to SiO_2_ CC with a mixed eluent of benzene and acetone (200 : 1) and further recrystallization of the minor fractions with chloroform/methanol to yield betulinic acid (**6**) (30 mg) and labda-8(17),13E-dien-15,18-dioic acid 15-methyl ester (**7**) (5 mg), respectively. F5.8 was recrystallized with chloroform/methanol to produce mixture of *β*-sitosterol (**8**) and stigmasterol (**9**) (630 mg). F5.10 was repeatedly subjected to SiO_2_ CC and pTLC (eluted with benzene : acetone, 20 : 1) to yield *ψ*-taraxasterol (**10**) (8 mg).

Fraction 8 was subjected to SiO_2_ CC eluted with chloroform/methanol gradients (50 : 1 to 1 : 1) and monitored by TLC to afford five subfractions (F8.1~8.5). Subfraction F8.1 was further recrystallized with chloroform/methanol to yield betulinic acid (**6**) (2 mg). F8.2 was repeatedly subjected to SiO_2_ CC and pTLC (eluted with chloroform : methanol, 50 : 1) to afford *ψ*-taraxasterol (**10**) (2 mg) and 3*β*-hydroxyanticopalic acid (**11**) (12 mg), respectively. The subfraction F8.3 was purified with SiO_2_ CC eluted with chloroform and methanol gradients (50 : 1 to 1 : 1) to yield three minor fractions (F8.3.1~8.3.3). The minor fraction F8.3.2 was further applied to pTLC eluted with benzene/acetone (10 : 1) to yield agatholic acid (**12**) (9 mg). F8.3.3 was repeatedly subjected to SiO_2_ CC (eluted with chloroform/acetone, 50 : 1) and pTLC (eluted with benzene/acetone, 30 : 1) to yield 3,4-dimethoxybenzoic acid (**13**) (6 mg). Subfraction F8.4 was applied to SiO_2_ CC eluted with chloroform and methanol gradients (50 : 1 to 1 : 1) to yield four minor fractions (F8.4.1~8.4.4). The minor fractions F8.4.2 and 8.4.3 were major fractions and displayed significant spots by TLC monitoring. F8.4.2 was further repeatedly subjected to SiO_2_ CC and pTLC (eluted with* n*-hexane/acetone, 1 : 1) to yield vanillic acid (**14**) (12 mg) and syringic acid (**15**) (3 mg). F8.4.3 was further recrystallized with chloroform/methanol to yield phillyrin (**16**) (30 mg). Subfraction F8.5 was purified by SiO_2_ CC eluted with chloroform and methanol gradients (50 : 1 to 1 : 1) to yield three minor fractions (F8.5.1~8.5.3). The minor fraction F8.5.1 was further repeatedly subjected to SiO_2_ CC and pTLC (eluted with chloroform/ethyl acetate, 10 : 1) to afford* p*-hydroxyphenylacetic acid (**17**) (10 mg). F8.5.2 was isolated by pTLC eluted with chloroform/acetone (4 : 1) to produce* p*-hydroxybenzoic acid (**18**) (15 mg). F8.5.3 was further recrystallized with acetone to yield benzoic acid (**19**) (16 mg).

The water extracts were applied to a reversed-phase Diaion HP-20 column eluted with water and methanol gradients to afford six fractions as monitored by C-18 TLC; however, no constituents were identified from fractions 1–3. Fraction 4 (wF4) was subjected to SiO_2_ CC eluted with chloroform/methanol gradients (100 : 1 to 1 : 1) and monitored by TLC to afford five subfractions (wF4.1~4.5). The subfraction wF4.1 was purified with SiO_2_ CC eluted with chloroform and acetone gradients (100 : 1 to 1 : 1) to yield* p*-hydroxyphenylacetic acid methyl ester (**20**) (5 mg). Subfraction wF4.2 was applied to SiO_2_ CC eluted with chloroform and acetone gradients (200 : 1 to 1 : 1) to yield four minor fractions (wF4.2.1~wF4.2.4). The minor fraction wF4.2.1 was further recrystallized with chloroform/methanol to afford* p*-tyrosol (**21**) (10 mg). The minor fractions wF4.2.2 and wF4.2.3 were further repeatedly subjected to SiO_2_ CC and pTLC (eluted with chloroform/methanol, 30 : 1) to afford* p*-hydroxybenzoic acid (**18**) (5 mg) and* p*-hydroxyphenylacetic acid (**17**) (4 mg), respectively. The minor fraction wF4.2.4 was subjected to SiO_2_ CC and further purified by pTLC (eluted with chloroform/methanol, 20 : 1) to yield hydroxytyrosol (**22**) (3 mg). Subfraction wF4.4 was subjected to SiO_2_ CC eluted with chloroform and acetone gradients (100 : 1 to 1 : 1) to yield five minor fractions (wF4.4.1~wF4.4.5). The minor fractions wF4.4.2, wF4.4.4, and wF4.4.5 displayed significant spots and were applied to SiO_2_ CC, eluted with chloroform/methanol (10 : 1) to afford 2-furancarboxylic acid (**23**) (15 mg), salidroside (**24**) (18 mg), and (6*S*,9*R*)-roseoside (**25**) (10 mg), respectively. Subfraction wF4.5 was repeatedly subjected to SiO_2_ CC (eluted with chloroform/methanol, 10 : 1) and further recrystallization of the minor fractions with chloroform/methanol to result in forsythoside D (**26**) (8 mg), methyl-*α*-D-glucopyranoside (**27**) (10 mg), and adoxosidic acid (**28**) (15 mg), respectively.

Fraction 5 (wF5) was subjected to SiO_2_ CC eluted with chloroform/methanol gradients (200 : 1 to 1 : 1) and monitored by TLC to afford five subfractions (wF5.1~5.5). Subfractions wF5.1, wF5.3, and wF5.4 displayed significant spots and therefore were subjected to the further purification. Subfraction wF5.1 was repeatedly subjected to SiO_2_ CC (eluted with chloroform/acetone, 300 : 1 to 1 : 1) and further recrystallized with chloroform/methanol to result in* p*-hydroxyphenylacetic acid methyl ester (**20**) (3 mg). Subfraction wF5.3 was applied to SiO_2_ CC (eluted with chloroform/acetone, 300 : 1 to 1 : 1) and further recrystallized with chloroform/methanol to yield* p*-hydroxyphenylacetic acid (**17**) (5 mg) and protocatechualdehyde (**29**) (5 mg). Subfraction wF5.4 was repeatedly purified by SiO_2_ CC (eluted with chloroform/acetone, 200 : 1 to 1 : 1) and further recrystallization of the minor fractions with chloroform/methanol to yield esculetin (**30**) (3 mg) and caffeic acid (**31**) (12 mg), respectively. Fraction 6 (wF6) was isolated by SiO_2_ CC eluted with chloroform/methanol gradients (100 : 1 to 1 : 1) and monitored by TLC to result in five subfractions (wF6.1~6.5). Only subfractions wF6.2 and wF6.3 displayed significant spots and therefore were subjected to the further purification. Subfraction wF6.2 was repeatedly purified by SiO_2_ CC (eluted with chloroform/acetone, 200 : 1 to 1 : 1) and further recrystallization of the minor fractions with acetone to yield* trans*-coumaric acid (**32**) (5 mg) and* trans*-ferulic acid (**33**) (5 mg). Subfraction wF6.3 was further recrystallized with acetone to result in quercetin (**34**) (45 mg).

#### 2.3.1. Spectral Data of **1**

White powder (CHCl_3_), mp 238–245°C; [*α*]_*D*_^25^−118.0 (*c* 0.09, CHCl_3_); IR (Neat) *ν*_max⁡_: 3442, 2948, 1760, 1727, 1444, 1375, 1250 cm^−1^; ^1^H NMR (CDCl_3_, 400 MHz) *δ* 0.83 (15H, m, CH_3_-23, 24, 25, 27, 29), 0.94 (3H, s, CH_3_-26), 1.35 (3H, s, CH_3_-30), 2.05 (3H, s, CH_3_-32), 2.10 (1H, m, H-15), 2.60 (2H, m, H-16), 4.48 (1H, dd,* J* = 10.4, 5.6 Hz, H-3*α*); ^13^C NMR (CDCl_3_, 125 MHz) *δ* 15.5 (C-26), 16.2 (C-23, 24, 25), 16.5 (C-27), 18.1 (C-6), 21.3 (C-32), 21.4 (C-11), 23.7 (C-2), 25.0 (C-12), 25.4 (C-30), 26.8 (C-22), 28.0 (C-29), 29.2 (C-16), 31.2 (C-15, 21), 35.1 (C-7), 37.1 (C-10), 37.9 (C-4), 38.7 (C-1), 40.4 (C-8), 43.2 (C-14, 18), 49.4 (C-13), 50.2 (C-17), 50.5 (C-9), 55.9 (C-5), 80.9 (C-3), 90.1 (C-20), 171.0 (C-31), 176.8 (C-28); FAB-MS* m/z *(*rel. int.*) 517 ([M+H]^+^, 100); HR-FAB-MS* m/z *517.3896 [M+H]^+^ (calcd for C_32_H_53_O_5_, 517.3893).

### 2.4. Antimicrobial Activity

#### 2.4.1. Microorganisms

The antimicrobial activity was evaluated against* Escherichia coli* (BCRC-11634). The strains were kept at −70°C in Luria-Bertani agar (LBA), activated by transferring into nutritive agar and incubating at 37 ± 1.0°C for 18 h. The bacterial suspension of each strain was prepared in a sterile tube with glass pearls and turbidity adjusted with distillated water, according to McFarland scale number 1 tube, which corresponds to approximately 3 × 10^8^ CFU/mL [13].

#### 2.4.2. Determination of the In Vitro Antimicrobial Activity

The antimicrobial activities against* E. coli* of different concentrations of tested samples were determined by the microtiter plate method described by the United States Pharmacopeia [17]. A twofold microdilution broth method was used to determinate the minimum inhibitory concentrations (MIC) value for each test substance [18–21]. Each well contained 10^6^ CFU/mL of test bacteria and LB medium (100 *μ*L). 100 *μ*L of MeOH solutions of tested samples (5 mg/mL for pure compounds and 20 mg/mL for the fractions) was added to wells of the first row. Dilutions were used to dispense 100 *μ*L into the other sterile 96 wells of an ELISA plate using a multichannel micropipette, resulting in eight concentrations to be tested for each compound. A negative control containing inoculated growth medium and methanol was prepared. Each experiment was performed in triplicate.

#### 2.4.3. Minimum Inhibitory Concentration (MIC) Determination

The MIC value is a measure to define the antibacterial activity of a compound and is defined as the lowest concentration of drug that inhibits visible growth. The amount of growth in the wells containing test samples was compared with the amount of growth in the control wells when determining the growth end points. When a single skipped well occurred, the highest MIC was read.

## 3. Results and Discussion

### 3.1. Isolation and Characterization of Compounds

**Figure 1:**
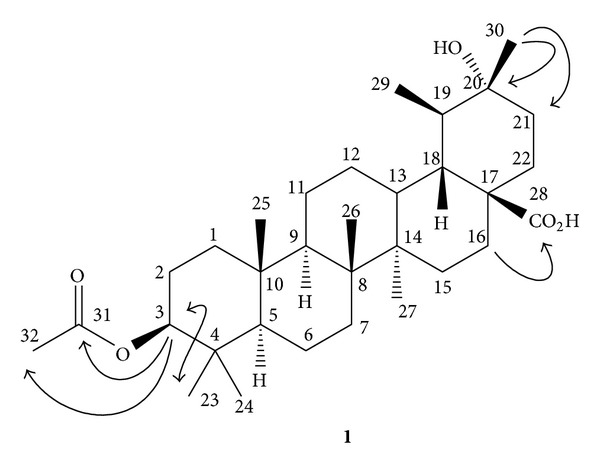
Chemical structure, significant HMBC (→) and NOESY (*↔*) correlations of compound** 1**.

Dried and powdered fruits of* F. suspensa *were extracted with methanol, and the combined extracts were concentrated under reduced pressure to give deep brown syrup. The crude extract was suspended into water and partitioned with chloroform to afford chloroform and water soluble fractions, respectively. Purification of the chloroform fraction of the methanol extracts of fruits of* F. suspensa* by a combination of chromatographic techniques yielded one new triterpene, 3*β*-acetoxy-20*α*-hydroxyursan-28-oic acid (**1**) (Figure 1), *β*-amyrin acetate (**2**) [22], taraxasterol acetate (**3**) [23], 3*β*-acetyl-20,25-epoxy-dammarane-24*α*-ol (**4**) [24], acetyl oleanolic acid (**5**) [25], betulinic acid (**6**) [26], labda-8(17),13*E*-dien-15,18-dioic acid 15-methyl ester (**7**) [27], mixture of *β*-sitosterol (**8**) and stigmasterol (**9**) [28], *ψ*-taraxasterol (**10**) [29], 3*β*-hydroxyanticopalic acid (**11**) [30], agatholic acid (**12**) [31], 3,4-dimethoxybenzoic acid (**13**) [32], vanillic acid (**14**) [33], syringic acid (**15**) [33], phillyrin (**16**) [15],* p*-hydroxyphenylacetic acid (**17**) [34],* p*-hydroxybenzoic acid (**18**) [33], and benzoic acid (**19**) [35], respectively. The water fraction was subjected to the reversed-phase Diaion HP-20 column chromatography and successive isolation to afford* p*-hydroxyphenylacetic acid (**17**),* p*-hydroxybenzoic acid (**18**),* p*-hydroxyphenylacetic acid methyl ester (**20**) [36],* p*-tyrosol (**21**) [37], hydroxytyrosol (**22**) [38], 2-furancarboxylic acid (**23**) [39], salidroside (**24**) [40], (6*S*,9*R*)-roseoside (**25**) [41], forsythoside D (**26**) [8], methyl-*α*-D-glucopyranoside (**27**) [42], adoxosidic acid (**28**) [43], protocatechualdehyde (**29**) [44], esculetin (**30**) [45], caffeic acid (**31**) [46],* trans*-coumaric acid (**32**) [47],* trans*-ferulic acid (**33**) [48], and quercetin (**34**) [49], respectively. The chemical structures of known compounds** 2–34** were identified by comparison of their physical and spectroscopic data with those reported in the literature. Among the isolates, compounds** 2**,** 4**,** 6**,** 8**,** 14**,** 16**,** 17**,** 24**,** 26**,** 28**,** 31**, and** 34** had been identified from the titled plant. Other compounds were reported from* F. suspensa* for the first time. Compound** 1** was a new compound and its structure was established by the spectral analysis.

### 3.2. Structural Elucidation of Compound **1**

The purified white powder** 1** was visualized by spraying with 1% (w/v) Ce(SO_4_)_2_ in 10% (v/v) aqueous H_2_SO_4_ followed by heating at 120°C and displayed purplish black spots on TLC plate. It also displayed positive responses against the Lieberman-Burchard test. These results suggested compound** 1** to be a triterpenoid [50]. The molecular formula of** 1** was established as C_32_H_52_O_5_ by the pseudomolecular [M+H]^+^ ion peak at* m/z* 517.3896 in HR-FAB-MS analysis and was further supported by its ^13^C-NMR spectrum which showed signals for all the 32 carbons of the molecule including one set of acetyl group (*δ*_C_ 171.0, 21.3), one carboxylic acid group (*δ*_C_ 176.8), one oxygenated quaternary carbon (*δ*_C_ 90.1), and one acetoxygenated carbon (*δ*_C_ 80.9), respectively. In the ^1^H-NMR spectrum of** 1**, there were proton signals for seven methyl groups at *δ* 0.83 (15H, m, and CH_3_-23, -24, -25, -27, and -29), 0.94 (3H, s, and CH_3_-26), and 1.35 (3H, s, and CH_3_-30), and one acetyl methyl group at *δ* 2.05 (3H, s, and CH_3_-32), respectively. The spectroscopic data indicated compound** 1** to possess oleanane type basic skeleton. In the downfield region, one oxygenated proton at *δ* 4.48 (1H, dd,* J* = 10.4, 5.6 Hz, H-3*α*) was located at C-3 which was further established by the NOESY correlations between CH_3_-23 and H-3. The ^2^ *J*, ^3^ *J*-HMBC correlations from *δ* 4.48 (H-3) to *δ*_C_ 21.3 (C-32) and 171.0 (C-31) also evidenced the presence of acetoxy group at C-3. The substitution of tertiary alcohol at C-20 was also determined with the HMBC analysis of correlations from CH_3_-30 to C-21 (*δ*_C_ 31.2) and C-20 (*δ*_C_ 90.1). The ^2^ *J*, ^3^ *J*-HMBC correlation peak between *δ* 2.60 (m, H-16) and *δ*_C_ 176.8 (C-28) supported the carboxylic acid group to be attached at C-17. The complete assignments of ^1^H and ^13^C NMR signals of** 1** were furnished from the NOESY and HMBC spectra. Therefore the chemical structure of** 1** was established as 3*β*-acetoxy-20*α*-hydroxyursan-28-oic acid and shown in Figure 1.

### 3.3. The Antimicrobial Effects of Isolated Compounds against* Escherichia coli*

**Figure 2:**
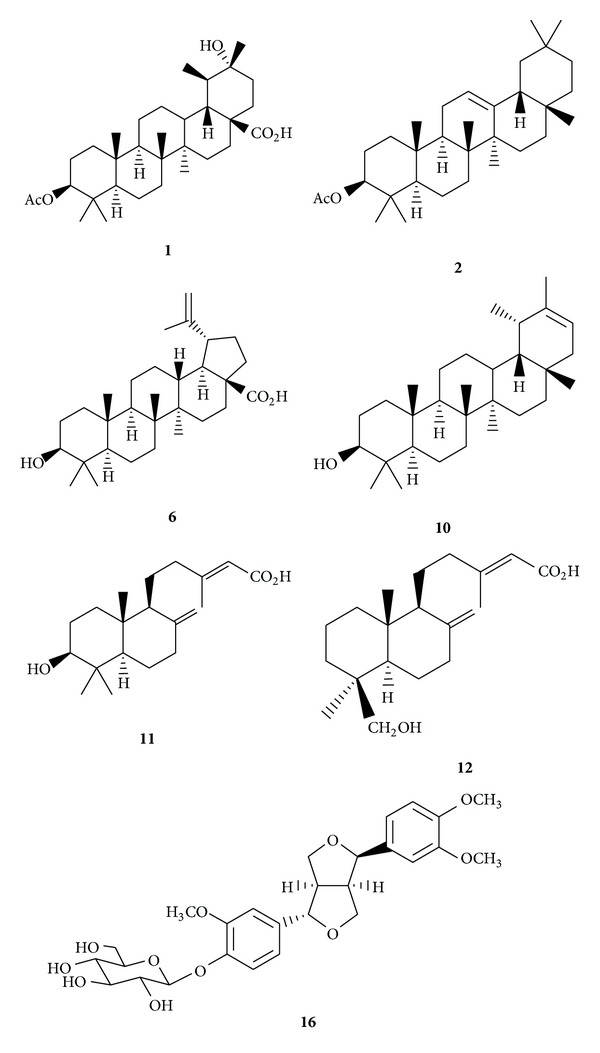
Structures of the isolated compounds subjected to the antimicrobial assay.

The crude extracts, partially purified fractions, and some of the purified principles (Figure 2) were subjected to the examinations for the inhibitory effects against* E. coli* [17–21]. The MIC data of the fractions were presented in Table 1. The MIC value of crude extracts (FS) was 4.25 mg/mL and demonstrated inhibition of the bacterial growth. Comparatively, the chloroform fraction (FSC) displayed more significant inhibitory effects against* E. coli* (BCRC-11634) than the water fraction (FSW) with MIC values of 6.25 and 12.50 mg/mL, respectively. When studying the influence of the concentration of compounds on the antimicrobial activities against* E. coli*, twofold microdilution broth method was used for the purified principles from the chloroform fraction (FSC), including triterpenoids** 1**,** 2**,** 6**, and** 10**; diterpenoids** 11**and** 12**; and lignan** 16**. It was observed that as the concentration increased, the inhibition of the bacterial growth was also increased. All of the tested samples demonstrated the inhibitory effects in a concentration-dependent manner. The MIC data of the examined compounds were presented in Table 2. The MIC values were in the range between 1.20 and 5.00 mg/mL against* E. coli* (BCRC-11634). Among the tested compounds, triterpenoids betulinic acid (**6**) and *ψ*-taraxasterol (**10**) exhibited the most significant inhibition against* E. coli* with MIC values of 1.20 mg/mL. These principles should be responsible for the bioactivity of the chloroform fraction (FSC). The results exhibited that the triterpenoids from the methanol extracts of fruits of* F. suspensa* possessed antibacterial activities against the common bacteria. It also provided evidence for the traditional uses of the fruits of* F. suspensa* as herbal medicines in the treatment of bacterial diseases. Although these purified compounds did not display better inhibition of the bacterial growth compared with the reported synthetic antibiotics, the extracts and principles from the natural sources usually possessed lower toxicity. Further structural modification could be performed to improve the activity and maintain the safety of these compounds. Therefore, it would be potentially useful in developing new antimicrobial therapeutic agents.

## Supplementary Material

The 1D and 2D NMR spectra of the new compound (**1**) were provided.

## Figures and Tables

**Table 1:** The minimum inhibitory concentrations (MICs) of the crude extract and partial purified fractions of *F. suspensa* against *E. coli* (BCRC-11634).

Sample	MIC (mg/mL)
FS (crude extracts)	4.25
FSC (chloroform fraction)	6.25
FSW (water fraction)	12.50

**Table 2:** The minimum inhibitory concentrations (MICs) of the purified samples from *F. suspensa* against *E. coli* (BCRC-11634).

Compound	MIC (mg/mL)
**1**	4.55
**2**	5.00
**6**	1.20
**10**	1.20
**11**	3.42
**12**	2.62
**16**	3.94
