# Supplementary material for: Chemical Constituents from the Fruits of Forsythia suspensa and Their Antimicrobial Activity
Source: Biomed Res Int. 2014 Mar 12;2014:304830. doi: 10.1155/2014/304830 (PMC3972829; doi:10.1155/2014/304830)

**SUPPORTING INFORMATION**

**Chemical Constituents from the Fruits of *Forsythia suspensa* and Their Antimicrobial Activity**

**Ping-Chung Kuo,^1,^* Guo-Feng Chen,^1,2^ Mei-Lin Yang,^2^ Ya-Hua Lin,^1^ and Ji-Jhong Peng^1^**

^1^ *Department of Biotechnology, National Formosa University, Yunlin 632, Taiwan, ROC*

^2^ *Department of Chemistry, National Chung-Hsing University, Taichung 402, Taiwan, ROC*

* Corresponding should be addressed to Dr. Ping-Chung Kuo, pcckuoo@nfu.edu.tw. Tel.: +886-5-6315491; fax: +886-5-6315502.

**Supporting Information Contents**

**S1.** The ^1^H NMR spectrum of **1** (CDCl_3_, 400 MHz).

**S2.** The ^13^C and DEPT135 NMR spectrum of **1** (CDCl_3_, 125 MHz).

**S3.** The COSY spectrum of **1**.

**S4.** The NOESY spectrum of **1**.

**S5.** The HMQC spectrum of **1**.

**S6.** The HMBC spectrum of **1**.

**S1.** The ^1^H NMR spectrum of **1** (CDCl_3_, 400 MHz).


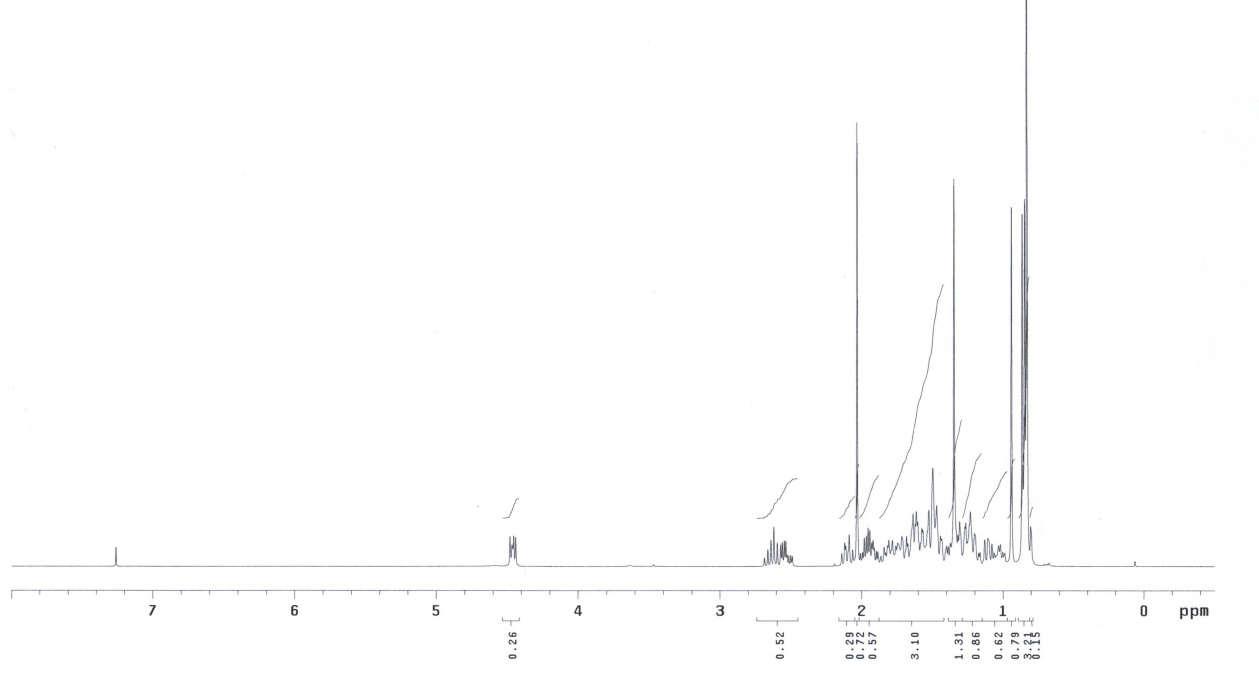


**S2.** The ^13^C and DEPT135 NMR spectrum of **1** (CDCl_3_, 125 MHz).


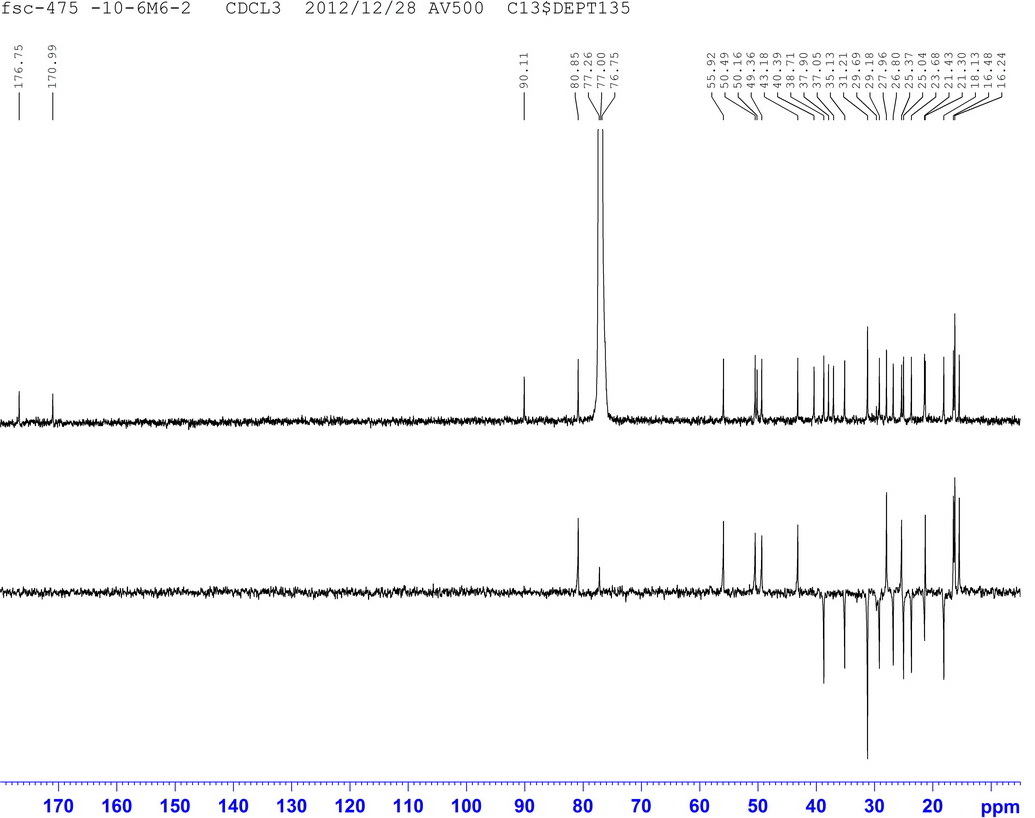


**S3.** The COSY spectrum of **1**.


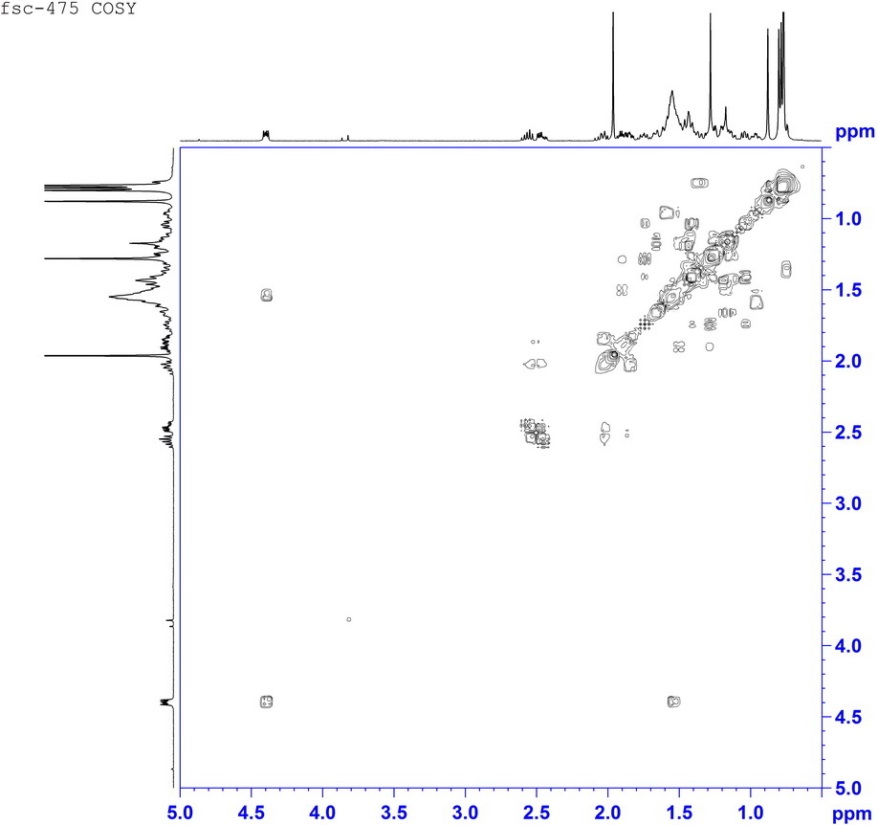


**S4.** The NOESY spectrum of **1**.


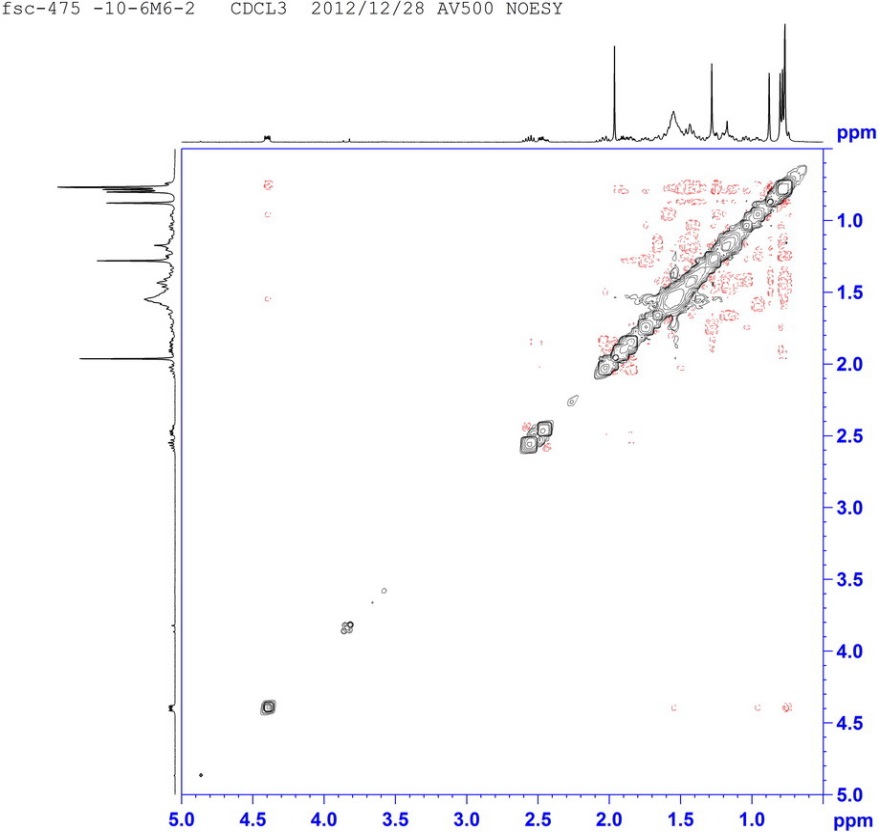


**S5.** The HMQC spectrum of **1**.


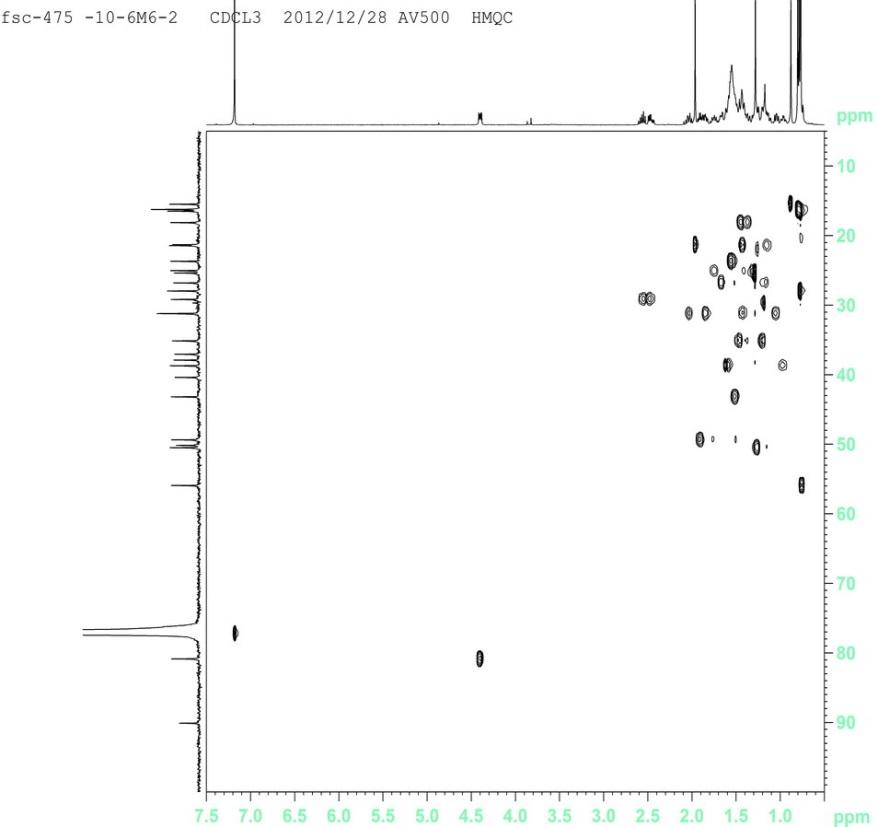


**S6.** The HMBC spectrum of **1**.


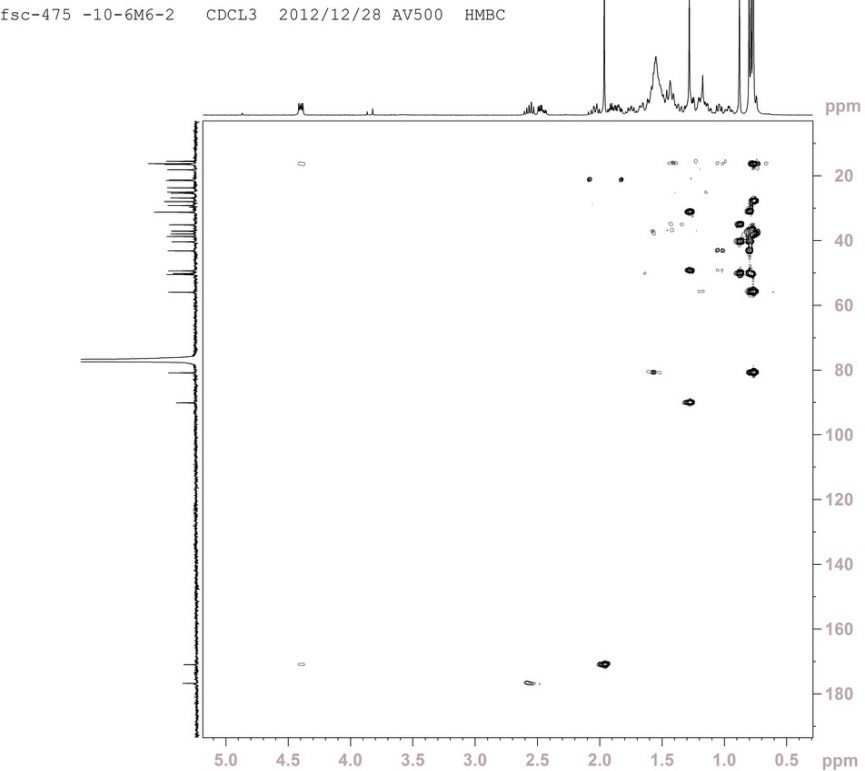

Supplement: Supplementary file 1 — The 1D and 2D NMR spectra of the new compound (1) were provided. [file 304830.f1.docx]
